# Human Activity Recognition with Noise-Injected Time-Distributed AlexNet

**DOI:** 10.3390/biomimetics10090613

**Published:** 2025-09-11

**Authors:** Sanjay Dutta, Tossapon Boongoen, Reyer Zwiggelaar

**Affiliations:** Department of Computer Science, Aberystwyth University, Ceredigion SY23 3DB, UK; tob45@aber.ac.uk (T.B.); rrz@aber.ac.uk (R.Z.)

**Keywords:** human activity recognition, AlexNet, noise injection, regularisation techniques, deep learning

## Abstract

This study investigates the integration of biologically inspired noise injection with a time-distributed adaptation of the AlexNet architecture to enhance the performance and robustness of human activity recognition (HAR) systems. It is a critical field in computer vision which involves identifying and interpreting human actions from video sequences and has applications in healthcare, security and smart environments. The proposed model is based on an adaptation of AlexNet, originally developed for static image classification and not inherently suited for modelling temporal sequences for video action classification tasks. While our time-distributed AlexNet efficiently captures spatial and temporal features and suitable for video classification. However, its performance can be limited by overfitting and poor generalisation to unseen scenarios, to address these challenges, Gaussian noise was introduced at the input level during training, inspired by neural mechanisms observed in biological sensory processing to handle variability and uncertainty. Experiments were conducted on the EduNet, UCF50 and UCF101 datasets. The EduNet dataset was specifically designed for educational environments and we evaluate the impact of noise injection on model accuracy, stability and overall performance. The proposed bio-inspired noise-injected time-distributed AlexNet achieved an overall accuracy of 91.40% and an F1 score of 92.77%, outperforming other state-of-the-art models. Hyperparameter tuning, particularly optimising the learning rate, further enhanced model stability, reflected in lower standard deviation values across multiple experimental runs. These findings demonstrate that the strategic combination of noise injection with time-distributed architectures improves generalisation and robustness in HAR, paving the way for resource-efficient and real-world-deployable deep learning systems.

## 1. Introduction

Human activity recognition (HAR) is a challenging yet critical area in computer vision that enables the automated identification and interpretation of human actions from various data modalities, including visual data, sensor information and multimodal inputs [1,2]. The complexity of HAR arises from the need to process both spatial and temporal information in dynamic, real-world environments where human behaviours exhibit significant variability across contexts, individuals and environmental conditions [3,4]. As a foundational technology, HAR has found widespread applications across diverse domains, including healthcare monitoring, security surveillance, smart environments and assistive technologies, making it one of the most actively researched areas in computer vision and machine learning.

The significance of HAR extends beyond technical achievement to practical impact on human welfare and societal applications. In healthcare, HAR systems enable continuous monitoring of patient activities for rehabilitation assessment and fall detection. In security applications, these systems provide automated surveillance capabilities for identifying suspicious behaviours. Smart home environments leverage HAR for context-aware automation, while assistive technologies use activity recognition to support individuals with disabilities or age-related limitations.

In recent years, HAR has gained growing attention in educational contexts, where understanding student behaviour can offer valuable insights into engagement, participation and overall learning dynamics. In classroom environments, recognising activities such as hand-raising, note-taking, reading or mobile phone use enables educators and intelligent tutoring systems to assess behavioural patterns, identify distraction or disengagement and tailor interventions accordingly. The integration of HAR into learning analytics frameworks can enhance real-time feedback mechanisms, promote adaptive teaching strategies and contribute to the development of inclusive, student-centered classrooms. This educational application of HAR represents a particularly promising frontier, as it combines the technical challenges of activity recognition with the potential for meaningful educational impact.

This study focuses specifically on HAR within classroom settings, leveraging video data from the EduNet dataset to detect student behaviours during instructional activities. By advancing robust and generalisable HAR models tailored for educational environments, this work aims to bridge the gap between computer vision techniques and practical applications in technology-enhanced learning. The educational context presents unique challenges, including environmental noise, occlusions and variability in classroom behaviours, which require specialised approaches to achieve reliable performance.

In this research, we focus on the application of the time-distributed AlexNet model, a novel architecture inspired by the original AlexNet model [5], which was originally designed for static image classification and lacks the capability to handle temporal sequences. Our adapted model efficiently captures spatial features across individual video frames while indirectly preserving temporal dependencies [6], without explicit use of temporal layers like LSTM, through sequence-level feature aggregation. This model is particularly suited for video-based HAR tasks where the preservation of spatial integrity over time plays a crucial role in achieving high recognition accuracy. Previous studies have demonstrated the significance of time-distributed layers in maintaining spatial consistency across temporal sequences, which is crucial for effective recognition in video datasets [7].

To evaluate the efficacy of the time-distributed AlexNet, we initially applied the model to the EduNet dataset, a well-structured dataset designed for HAR benchmarking in educational environments. The model demonstrated promising performance, effectively recognising a diverse range of human activities. However, real-world scenarios often introduce additional complexities such as environmental noise, occlusions and viewpoint variations, which can degrade model performance [8].

Our research explores the integration of biologically inspired principles into the design of an HAR model, emphasising noise-induced robustness and hierarchical feature extraction. These principles, inspired by the human visual system’s ability to process complex stimuli under varying conditions, equip the model to effectively address real-world challenges [9]. Experimental evaluations on the benchmark dataset demonstrate the model’s state-of-the-art performance, validating its applicability to real-world educational problems.

A key contribution of this research is its focus on addressing significant HAR challenges, such as noise resilience and temporal dynamics in educational settings. By incorporating biologically inspired noise injection, the study advances methodologies in vision-based systems, creating more robust and adaptable HAR frameworks specifically tailored for classroom applications. This approach draws inspiration from biological visual systems, such as those found in dipteran flies, which demonstrate remarkable robustness in processing complex visual information under varying environmental conditions. Research has shown that fly visual systems effectively utilise chromatic and achromatic processing mechanisms to discriminate between different stimuli despite environmental variations, noise and changing illumination conditions [10]. Similarly, the human visual system exhibits exceptional noise tolerance and adaptive processing capabilities when interpreting complex visual scenes [11]. By emulating these biological principles of noise resilience and adaptive feature processing, our HAR model incorporates structured noise injection to simulate the natural variability encountered by biological visual systems, thereby enhancing the model’s ability to maintain performance consistency across diverse classroom environments with varying lighting conditions, occlusions and visual perturbations.

Noise injection is a well-established regularisation technique [12] that plays a central role in this approach. While Gaussian noise [13] has been explored in various deep learning contexts, its structured integration within a time-distributed AlexNet framework specifically for video-based HAR remains underexplored. In this study, we contribute a novel design by incorporating Gaussian noise directly into the temporal feature stream, enabling the network to encounter subtle variations across frames in sequence, thereby enhancing temporal robustness. By perturbing input data with Gaussian noise to augment the EduNet dataset, the model is trained to simulate real-world variations. This method enhances the robustness and generalisation of the time-distributed AlexNet, enabling improved recognition of activities under challenging conditions while mitigating overfitting.

**Key Contributions:** This study made the following specific contributions to the field of HAR:

- We introduced a time-distributed adaptation of the AlexNet architecture, which was originally developed for static image classification, to support temporal feature extraction in video-based HAR tasks.
- We integrated biologically inspired Gaussian noise injection at the input stage of the model to enhance robustness and generalisation, simulating sensory variability and improving resilience to real-world visual perturbations.
- We systematically explored and fine-tuned the noise injection hyperparameter across 17 values and demonstrated, through statistical validation, that an optimal noise level (standard deviation = 0.01) improved model performance while reducing overfitting.
- We trained and evaluated state-of-the-art models such as ViT and ConvLSTM on the EduNet dataset.
- Additionally, our proposed method is evaluated on the UCF50 and UCF101 datasets.
- Our study provided practical insights into noise-driven regularisation for temporal deep models and presented an open, reproducible pipeline applicable to real-world educational environments where variability in lighting, occlusion and camera motion are common.

The rest of this paper is organised as follows. Section 2 provides a review of the technological development on HAR. After that, Section 3 presents the proposed method, including details of the time-distributed AlexNet architecture and the noise injection as the regularisation method. Then, Section 4 includes the experimental design and results that cover a comparison between the proposed methods and other state-of-the-art techniques. Discussion of challenges, limitations and future research directions is highlighted in Section 5 before the paper is concluded in Section 6.

## 2. Literature Review

### 2.1. Traditional Applications of Noise Injection

Noise injection has been established as a fundamental regularisation technique in neural networks since the work of Bishop [14], who demonstrated the theoretical connection between training with noise and Tikhonov regularisation. This foundational work showed that adding noise to inputs during training is mathematically equivalent to imposing a penalty on the squared magnitude of the first derivatives of the network function, thereby encouraging smoother decision boundaries and improved generalisation.

The regularisation effect of Gaussian noise injection (GNI) has been further analysed through explicit regularisers derived by marginalising out the injected noise [15]. These studies reveal that GNI penalises functions with high-frequency components in the Fourier domain, particularly in layers closer to the network’s output, which helps explain its effectiveness in reducing overfitting.

The paper [16] introduced a new class of probabilistic activation functions for neural networks that improved generalisation by injecting and adaptively optimising the level of noise during training—a process inspired by stochastic resonance. This approach enabled the design of activation functions (such as GEU and PGELU) with tunable noise parameters, which were shown experimentally and theoretically to improve generalisation and robustness, especially in the presence of noise compared to traditional activation functions, on regression and image classification tasks. Theoretical analysis linked the noise to reduced network complexity (via Rademacher complexity), which explained the improved generalisation.

Feng et al. [17] provided a theoretical framework for understanding noise injection in Generative Adversarial Networks (GANs), particularly as used in StyleGAN. The authors identified an “adversarial dimension trap” where GANs struggled because the generator’s expressive power was limited by the rank of its Jacobian matrix, which decreased monotonically with network depth, making it difficult to capture high-dimensional data manifolds. They proposed that noise injection effectively addressed this limitation by allowing generators to map onto low-dimensional “skeleton” sets of feature manifolds and use random noise to fill the remaining space, thereby avoiding the need to directly model higher-dimensional manifolds. Using Riemannian geometry, they formalised this process through exponential maps and developed a generalised Riemannian Noise Injection (RNI) method that extended beyond the simple Euclidean noise injection used in StyleGAN. Experiments on the FFHQ, LSUN and CIFAR-10 datasets validated their theory, showing improved image quality metrics (FID, PPL) and better numerical stability compared to standard approaches.

Ferianc et al. [18] systematically evaluated various noise injection methods—such as MixUp, Dropout, AugMix during neural network training, focusing on their effects on generalisation (how well models perform on unseen data) and confidence calibration (how well predictions’ probabilities reflect true outcomes) across diverse tasks, datasets and architectures. The study found that noise generally improved either generalisation or calibration and, in some cases, both. The optimal methods varied by task: AugMix and weak augmentation were best for computer vision, Dropout for NLP and Gaussian weight noise for tabular regression. Combining two well-chosen noise types often gave further improvements, mostly for classification. However, the best hyperparameters for one domain or architecture often did not transfer well to others, so task-specific tuning remains important. Overall, noise injection can reduce overfitting and overconfidence, but simultaneous gains in accuracy and calibration require careful, context-sensitive selection and tuning.

### 2.2. HAR in Applications and Evaluations

HAR in educational settings presents unique challenges due to environmental noise, occlusions and variability in classroom behaviours. Previous research has explored a range of deep learning models to address HAR tasks, achieving high accuracy on controlled datasets. However, many existing approaches lack robustness to real-world variability and limited attention has been given to biologically inspired regularisation strategies. This motivates our study, which integrates temporal modelling and noise resilience in a unified framework.

The literature reveals a wide spectrum of advancements in activity recognition, spanning classroom-specific and broader HAR contexts. Deep learning models, including Convolutional Neural Networks (CNNs), Long Short-Term Memory networks (LSTMs) and hybrid architectures, have enhanced classification accuracy and generalisation capabilities.

Classroom-based studies have primarily focused on recognising student emotions, engagement levels and teacher behaviours. Techniques such as Graph Convolutional Networks (GCNs) and Spatiotemporal CNNs have demonstrated notable success, achieving accuracies as high as 97.92% [19,20]. Biologically inspired approaches, like Motion Detection Neurons (MDN), further emphasise the utility of noise resistance and enhanced motion detection accuracy in activity recognition tasks [21].

In the broader HAR domain, hybrid models such as CNN-BiLSTM-BiGRU have effectively leveraged sensor data, achieving 99.32% accuracy on the WISDM dataset [22]. Structured noise injection has emerged as a novel technique for improving the training of neural networks, offering both enhanced classification accuracy and biological plausibility [23].

Video-based HAR studies have explored spatiotemporal architectures like LRCN-TDL and ConvLSTM, with the former achieving an impressive 93.6% accuracy on the UCF50 dataset [24]. The use of models like 3D CNNs has shown promise in educational settings, achieving competitive accuracy on the EduNet dataset [25]. Techniques such as Faster R-CNN and Adaboost have further extended HAR capabilities by addressing student behaviours and e-learning tasks with high precision and efficiency [26,27].

Table 1 provides a summary of the key studies reviewed, detailing the deep learning models or methods used, the year of publication, novel aspects of each approach, the datasets utilised and the results or accuracy achieved. These advancements underline the integration of spatiotemporal feature extraction, multimodal data handling and biologically inspired methodologies into different areas of applications.

While the surveyed studies achieved notable success in activity recognition, few works explicitly address robustness to noise and dynamic variability during training. Most prior approaches primarily rely on traditional regularisation techniques. Batch Normalisation stabilises learning by reducing internal covariate shift, while Dropout randomly deactivates neurons during training to prevent co-adaptation. L2 regularisation (weight decay) penalises large weight values to encourage simpler models. However, these techniques mainly regularise model complexity rather than directly enhancing robustness against input variability. Recent advances in biologically inspired noise injection [28] suggest that introducing controlled stochastic perturbations at the input level can train models to become more resilient to real-world variations. For instance, neural models trained to replicate blue–green chromatic opponency in biting flies have shown that certain input-level noise or contrast enhancements can significantly alter perceptual outcomes, highlighting the biological role of sensory variability in improving detection robustness [10]. Motivated by these insights, our study proposes a time-distributed AlexNet architecture combined with Gaussian noise injection at the input stage, aiming to improve generalisation and robustness against environmental variability in educational HAR tasks.

**Table 1:** Comprehensive summary of key studies.

Model/Method	Year	Novel Aspects	Dataset	Result/Accuracy
Deep Learning and Cloud Computing [29]	2018	Response to nonverbal cues in real time	Classroom multimodal data	Training Mode: 4405 ms, Presentation Mode: 1869 ms
Deep Spatiotemporal Features and ELM [30]	2019	Instructor activity recognition	IAVID-1, MuHAVI, IXMAS datasets	Superior accuracy
CNN and Mood Classification [31]	2019	Classroom mood analysis	NIT Karnataka classroom videos	87.65% accuracy
Faster R-CNN [26]	2020	Analyses student behaviors like hand-raising	Custom behavior corpus	57.6% mAP
Webcam-Based System [32]	2020	Detects nonverbal student behaviors	Classroom task videos	86.87% accuracy
DCGANSAR [33]	2020	Recognises student actions with GANs	Self-built dataset	10% accuracy improvement
Microsoft Emotion Recognition API [34]	2020	Recognises student emotions from facial expressions	University student videos	Significant emotional state shifts
Facial Recognition and IoT [35]	2021	Automated attendance system	Classroom images	89% accuracy
3D BP-TBR [36]	2021	Recognises teacher behavior using bilinear pooling	Custom and public datasets	94.45% accuracy
I3D-ResNet-50 [37]	2021	Enhances human activity recognition in classrooms	EduNet	72.3% accuracy
Multiple CNNs [38]	2021	Recognises head postures for student behavior	Real classroom surveillance	84% accuracy
OpenPose and DNN [39]	2021	Recognises student behavior with skeleton pose estimation	Classroom videos	Higher precision and recall
Adaboost Classifier [27]	2022	Analyses e-learning behaviors	MED and EduNet datasets	83.25–84.25% accuracy
CNN-10 with Skeleton Extraction [19]	2022	High-accuracy student behavior recognition	Classroom behavior images	97.92% accuracy
3D CNNs and Conv2DLSTM [40]	2022	Analyses teaching actions in classroom videos	Custom video dataset	Highest accuracy of 94%
Graph Convolutional Network (GCN) [20]	2023	Detects nonverbal teacher behaviors	Classroom video recordings	94.68%, 81.64%
ATGCN [41]	2023	Detects classroom actions with RFID data	RFID tags on desks	96.9% accuracy
3-D Virtual Class Visualisation [42]	2023	Enhances teacher feedback in tele-education	Pilot application	98.05%, 94.32%
CNN-BiLSTM-BiGRU [22]	2024	Hybrid model for sensor-based HAR	WISDM dataset	99.32% accuracy
Training-With-Noise (TWN) [23]	2024	Structured noise for improved training	Synthetic datasets	Improved generalisation
Motion Detection Neurons (MDN) [21]	2024	Biologically inspired motion detection	Binary, grayscale and color datasets	Enhanced noise resistance
Long-term Recurrent Convolutional Network with Time-Distributed Layer (LRCN-TDL) [24]	2024	Spatiotemporal data handling for video HAR	UCF50, HMDB51	93.6%, 88.9% accuracy
Neural Networks [43]	2024	Classifies instructional activities without audio	Annotated classroom videos	Over 80% accuracy
3D CNNs [25]	2024	Classroom activity recognition using EduNet	EduNet	83.5% accuracy
OpenPose and Py-Feat [44]	2024	Classifies student behavior in real time	Classroom video frames	90.80% accuracy

## 3. Proposed Methods

### 3.1. Time-Distributed AlexNet Architecture

**Figure 1:**
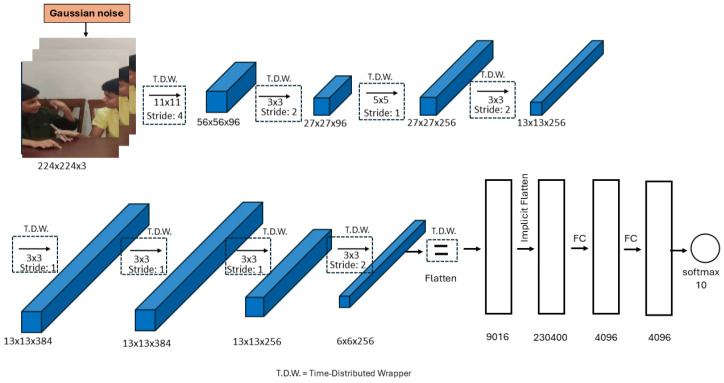
Architecture of noise injected time-distributed AlexNet [5].

The architecture of our model, shown in Figure 1, is based on the well-known AlexNet architecture [5], originally developed for static image classification. While standard AlexNet processes single RGB images, it lacks the capability to model temporal information, which is essential for video-based HAR.

To adapt AlexNet for sequence modelling, we introduced a TimeDistributed wrapper [45] around each convolutional and pooling layer. This wrapper applies the same set of operations independently to each frame in a video clip of fixed length while preserving the temporal order. Each input video is represented as a 5D tensor of shape (B,T,H,W,C), where *B* is the batch size, T=25 is the number of frames per clip, H=W=224 are the spatial dimensions and C=3 is the number of colour channels.

After convolutional processing, each frame is transformed into a feature vector of shape (9216) through a TimeDistributed(Flatten) operation. This yields a sequence of 25 frame-level features, resulting in a 3D tensor of shape (B,25,9216). To perform sequence-level classification, this sequence must be interpreted as a unified representation. While we did not explicitly insert an LSTM layer after the TimeDistributed(Flatten), the Keras Sequential model implicitly flattens this 3D tensor into a 2D tensor of shape (B,230400) when passed to the first Dense layer.

This flattening operation is equivalent to concatenating the 25 feature vectors along the temporal dimension and enables the Dense layers to model the entire video as a single spatiotemporal object. Importantly, this approach allows the model to capture temporal dependencies implicitly through the structure of the concatenated feature vector without requiring explicit temporal modules such as LSTMs or GRUs.

The model concludes with two fully connected layers, each with 4096 units and ReLU activations, interleaved with Batch Normalisation and Dropout [46] for regularisation. The final output layer is a Dense layer with 10 units (matching the number of activity classes), followed by a softmax activation for multi-class classification.

Table 2 summarises the architecture, including output dimensions and operations at each stage. In total, the model comprises 58,360,586 parameters, with 58,341,450 trainable and 19,136 non-trainable.

#### 3.1.1. Understanding Time-Distributed Layers

Time-distributed layers are designed to apply a layer (or a series of layers) to each time step of a sequence independently. This is particularly useful for processing sequential data such as video frames, where each frame is treated as a separate input. In the following, we will discuss how the time-distributed aspect works.

**Independent Processing of Each Frame:**

In a time-distributed layer, each frame of the video sequence is passed through the same convolutional operations independently. This means that the same set of filters and operations are applied to each frame, allowing the model to extract spatial features from each frame without mixing information across different time steps.

**Preservation of Temporal Sequence:**

By processing each frame independently, the temporal order of the frames is preserved. The output of the time-distributed layer is a sequence of feature maps, one for each frame, maintaining the original sequence order.

**Combining Spatial Features Over Time (Optional):**

After the time-distributed layers have processed each frame, the resulting sequence of feature maps can be further processed by layers that can capture temporal dependencies, such as recurrent layers [47] (e.g., LSTM or GRU) or temporal pooling layers. These subsequent layers can then learn patterns and dependencies across the sequence of frames.

For instance, consider a video sequence with *T* frames, where each frame has a dimension of H×W×C (height, width, channels). When using a time-distributed convolutional layer, the operation can be described as follows.

**Input**

The input to the time-distributed layer is a sequence of frames: $\left[X_{1},X_{2},\ldots ,X_{T}\right]$, where each $X_{i}$ is a frame of size H×W×C.

**Convolutional Operation**

For each frame $X_{i}$, the same convolutional layer with filters *W* is applied.

(1) $$Y_{i}=\mathrm{Conv}\left(X_{i},W\right)$$

This results in an output feature map $Y_{i}$ for each frame $X_{i}$.

**Output:**

The output of the time-distributed convolutional layer is a sequence of feature maps: $\left[Y_{1},Y_{2},\ldots ,Y_{T}\right]$, where each $Y_{i}$ has a dimension corresponding to the feature map size.

To better illustrate the core mechanism of our time-distributed design, we provide the following pseudocode that conceptually describes how each frame in a video sequence is processed independently using shared CNN weights. This demonstrates how spatial features are extracted frame by frame while maintaining temporal coherence. Algorithm 1 presents the pseudocode for the proposed Time-Distributed AlexNet model.
**Algorithm 1** Frame-wise feature extraction and sequence aggregation using time-distributed AlexNet.**Require:** Video sequence $V=\{F_{1},F_{2},\ldots ,F_{T}\}$, where $F_{t}$ is the $t^{\mathrm{th}}$ frame **Require:** CNN layers $L_{1},L_{2},\ldots ,L_{n}$ with shared weights across time
  1:**for** each frame $F_{t}$ in *V* **do**  2:   $x_{t}\leftarrow F_{t}$  3:   **for** each CNN layer $L_{i}$ in $\left\{L_{1},\ldots ,L_{n}\right\}$ **do**  4:     $x_{t}\leftarrow L_{i}\left(x_{t}\right)$ {Apply CNN layer with shared weights}  5:   **end for**  6:   $h_{t}\leftarrow \mathrm{Flatten}\left(x_{t}\right)$ {Flatten frame-wise feature map}  7:   Store $h_{t}$ into *H*  8:**end for**  9:$H_{\mathrm{concat}}\leftarrow \mathrm{Flatten}\left(H\right)$ {Flatten all time steps into one vector of shape (T·D)}10:$y\leftarrow \mathrm{Dense}\;\mathrm{Layers}(H_{\mathrm{concat}})$ {Pass to fully connected classifier}11:**return** *y* {Predicted activity class}


#### 3.1.2. Comparing with 3D Convolutions

In contrast, a 3D convolutional layer [48] processes the entire spatiotemporal volume at once, applying convolutional filters that span both spatial (height and width) and temporal (time) dimensions. While this approach captures spatiotemporal features directly, it comes with higher computational costs and complexity. Additionally, 3D convolutions require more memory and can be harder to train effectively due to the increased number of parameters.

By using time-distributed layers, the model effectively captures spatial features from each frame independently while preserving the temporal sequence, allowing subsequent layers to learn and exploit temporal patterns in the data.

### 3.2. Gaussian Noise Injection in Time-Distributed AlexNet for HAR

Noise injection techniques have gained significant attention in recent years for their effectiveness in enhancing the generalisation ability of deep learning models and reducing overfitting [49]. In this study, we incorporated a (GaussianNoise) layer prior to the first convolutional layer in the model. This design choice allows the model to simulate realistic variability across video frames, introducing controlled stochastic perturbations during training. Our implementation applies noise in a spatiotemporal context across frame sequences. This enables the model to learn noise-invariant temporal features, thereby improving its robustness to inter-frame variation and enhancing generalisation across diverse classroom activities.

Rather than simply relying on architectural complexity or traditional regularisation methods, our approach directly simulates sensory uncertainty at the input level. Through systematic experimentation with different noise levels, we demonstrate that moderate Gaussian noise can lead to improved generalisation and test performance, validating its utility as a lightweight and biologically inspired regularisation technique in HAR tasks.

The application of Gaussian noise in neural networks has a dual effect.

- Preventing Overfitting: The noise forces the network to generalise better by discouraging reliance on specific features in the training set.
- Robust Feature Learning: The network is encouraged to learn more robust patterns that are not highly sensitive to small variations in the input.

The goal of this study is to explore the effect of Gaussian noise on model performance, with specific emphasis on how different noise values influence the model’s ability to classify activities in the EduNet dataset accurately.

While Gaussian noise injection is a widely used regularisation technique, our application introduces a distinct novelty through its integration into a customised time-distributed AlexNet architecture for HAR. This approach draws inspiration from biological neural processes that inherently deal with sensory noise and uncertainty. Furthermore, we conducted a detailed empirical study with a range of noise values, demonstrating statistically significant improvements in generalisation and performance, especially at a noise level of 0.01. The strategic placement of noise at the input stage, along with rigorous tuning and validation, underscores the unique contribution of this work within the context of spatiotemporal HAR.

#### Incorporating Gaussian Noise into the Model

In this study, Gaussian noise is introduced at the input stage of the network, just before the first convolutional layer, using TensorFlow’s GaussianNoise layer. This noise serves as a regularisation technique, helping to improve the model’s generalisation by preventing overfitting. The noise is applied to the video frames, which are processed as sequences of images representing different classroom activities.

The model is structured as a time-distributed Convolutional Neural Network (CNN), where each input video is treated as a sequence of 25 frames. Each frame is resized to 224×224 pixels and normalised to values between 0 and 1. These frames form a sequence representing a single activity, which is then passed through several convolutional layers to extract spatial features.

The architecture consists of multiple convolutional layers, interspersed with Batch Normalisation and MaxPooling layers, followed by fully connected layers for classification. Gaussian noise is applied consistently across all frames (See Figure 1) in the sequence before any convolutional operations are performed. This is achieved by using the TimeDistributed wrapper, which ensures that the same convolutional operations are applied to each frame independently.

The Gaussian noise is injected as follows:

TimeDistributed(GaussianNoise(gaussian_noise_value),
input_shape=(SEQUENCE_LENGTH, IMAGE_HEIGHT, IMAGE_WIDTH, 3)),

Explanation of the Process:

1. Input: The input to the model consists of a sequence of frames $\left[X_{1},X_{2},\ldots ,X_{25}\right]$, where each frame $X_{i}$ has a dimension of 224×224×3 (height, width, channels). This represents one classroom activity in the video.
2. Noise Injection: Gaussian noise is injected into each frame independently. For each frame $X_{i}$, random noise drawn from a Gaussian distribution $N(0,\sigma^{2})$ is added to the frame.
  (2) $${\tilde{X}}_{i}=X_{i}+N\left(0,\sigma^{2}\right)$$
  where ${\tilde{X}}_{i}$ represents the noisy frame and σ is the standard deviation, or noise value, that controls the intensity of the noise.
3. Effect of Noise: This injection of noise introduces small perturbations to each frame in the sequence, thereby forcing the model to learn more robust features. By preventing the model from relying on specific pixel-level patterns, noise injection enhances the model’s ability to generalise better to unseen data.
4. Output: The output of the Gaussian noise layer is a sequence of noisy frames $\left[{\tilde{X}}_{1},{\tilde{X}}_{2},\ldots ,{\tilde{X}}_{25}\right]$, which are then passed to the subsequent layers, such as convolutional and pooling layers, for further feature extraction.

Tuning of Noise Value: The noise value, which determines the standard deviation of the Gaussian distribution, was an important parameter in this study. Various noise values were explored, ranging from 0.01 to 1.0. The study found that moderate noise levels, such as 0.01, provided the best trade-off between introducing variability and maintaining the integrity of the input data. Higher noise levels, such as 0.5 or 1.0, introduced excessive distortion, making it difficult for the model to learn meaningful features, which negatively impacted its performance.

By applying Gaussian noise at the input stage in this controlled manner, the model became more resilient to distortions commonly found in real-world video recordings, such as lighting changes, minor occlusions and background motion. This enhanced its ability to correctly classify various classroom activities under different conditions.

## 4. Performance Evaluation

### 4.1. Experimental Design

#### 4.1.1. Investigated Data

**Figure 2:**
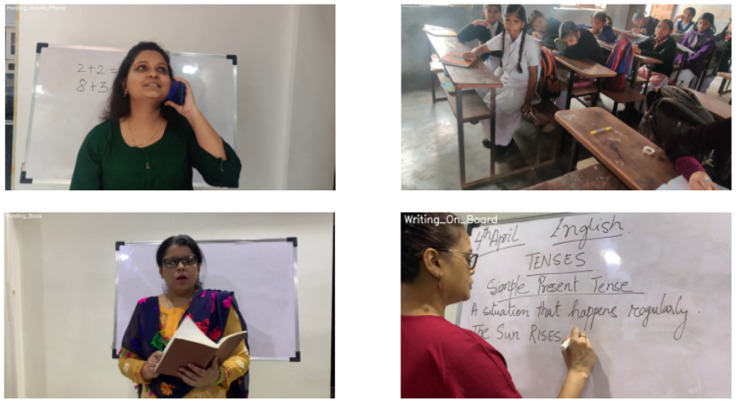
Examples from the EduNet dataset [37].

The EduNet dataset [37], trademarked under DRSTA™ and copyrighted in 2021, has been specifically tailored for advancing research in the field of artificial intelligence, with a focus on computer vision and HAR within educational settings. This subset version of the EduNet dataset comprises 10 distinct action classes (Table 3) that encompass a range of teacher and student activities observed in classroom environments. This collection comprises around 929 manually annotated video clips, drawn from both YouTube sources and direct recordings within actual classrooms. Although it is not clear how many samples are taken from YouTube, it ensures a rich and diverse dataset, as shown in Figure 2.

**Data Preprocessing:** The preprocessing pipeline involved several essential tasks to prepare the EduNet video dataset for classroom activity recognition using a deep learning model. All steps were implemented using Python and open-source libraries, including OpenCV, NumPy, TensorFlow/Keras and Scikit-learn.

Each video was uniformly sampled using a custom frame_extraction() function built with OpenCV’s cv2.VideoCapture, which extracts a fixed-length sequence of 25 frames to ensure temporal consistency across samples. Frames were selected at regular intervals throughout each video’s duration—regardless of its original frame rate—and resized to a resolution of 224×224 pixels using cv2.resize() to match the input dimensions required by the CNN model.

Pixel intensities were scaled to the range [0, 1] by dividing all values by 255.0 using NumPy arrays, helping normalise input values and accelerate convergence during training. Each video was annotated with one of ten predefined activity classes (see Table 3), such as “Arguing,” “Reading_Book,” and “Writing_On_Board.” These labels were encoded into one-hot categorical vectors using tensorflow.keras.utils.to_categorical() to support multi-class classification.

The complete dataset was split into training and testing subsets using an 80:20 ratio via Scikit-learn’s train_test_split(), with a fixed random seed to ensure reproducibility. Additionally, five-fold cross-validation was conducted using KFold from Scikit-learn to evaluate generalisation and validate the robustness and consistency of the model across different data splits. This comprehensive preprocessing pipeline ensures standardised formatting, consistent scaling and accurate labelling of input data, laying a solid foundation for effective model training and evaluation.

#### 4.1.2. Method-Specific Settings

To ensure optimal performance and prevent overfitting, we carefully tuned key hyperparameters and employed multiple regularisation techniques to improve generalisation to unseen classroom activity data. Table 4 summarises the key configuration used in the final Time-Distributed AlexNet model.

In our experiments, we fixed the number of frames per input video sequence to T=25. This value was selected to provide a sufficient temporal window to capture meaningful student activities such as reading, writing, or hand-raising while keeping memory usage feasible for training. Although the model architecture is scalable to longer sequences like T=50 or T=100, our preliminary trials showed diminishing returns in accuracy and increased training time due to redundant frames common in classroom scenarios.

The learning rate was tuned using a grid search over the range of 0.00005 to 0.001, with 0.0001 consistently yielding stable convergence and high validation accuracy (see Section 4.2.6 for statistical details). We applied Gaussian noise at the input layer to simulate real-world variability. Among the tested values (from 0.005 to 1.0), a standard deviation of σ=0.01 showed the best balance between performance and generalisation.

Batch Normalisation was applied after each convolutional layer to stabilise and accelerate training by reducing internal covariate shift. Dropout with a rate of 50% was added after fully connected layers to reduce overfitting. Early stopping was implemented with a patience of 15 epochs and training stopped at epoch 72 in the final run when no further validation improvement was observed. The best weights were restored for final evaluation.

#### 4.1.3. List of Compared Methods and Parameter Settings

To evaluate the performance of the proposed time-distributed AlexNet architecture, two additional state-of-the-art methods were selected for comparison. These methods were applied to the same EduNet dataset to ensure consistency in evaluation. The experimental setup was crucial in evaluating how different models process and learn from the temporal and spatial dynamics of classroom activities. Additionally, the comparison with other methods allowed us to highlight the improvements made by our approach, especially in handling raw video data without the need for manual feature extraction. The compared methods and their respective parameter settings are as follows.

1. **ViT (Vision Transformer) [50]**
  **Description:** This model adopts a transformer-based architecture for spatiotemporal modelling by applying patch-level embeddings across video frames. Each video is represented as a sequence of frame-wise patch vectors and temporal dependencies are captured using time-distributed transformer encoders.
**Parameter Settings:**
  - Input size: 224 × 224 frame dimensions with 16×16 patch extraction.
  - Patch embedding dimension: 128.
  - Transformer heads: Four multi-head self-attention heads used per encoder.
  - Learning rate: 0.001 with Adam optimiser.
  - Dropout rate: 50% applied before classification to prevent overfitting.
  - Sequence length: 25 uniformly sampled frames per video.
2. **ConvLSTM** [51]
  **Description:** This hybrid model combines convolutional layers for spatial feature extraction and LSTMs to capture temporal dependencies.
**Parameter Settings:**
  - Input size: 64 × 64 frame dimensions, scaled down for faster processing.
  - Learning rate: Default 0.001 with Adam optimiser.
  - Dropout rate: 20% was applied.

#### 4.1.4. Evaluation Method Used

- **K-Fold Cross-Validation:** During both the training–testing split and K-Fold cross-validation, splitting was performed strictly at the video level, using video file paths only. This guarantees that all frames from a given video belong exclusively to either the training or the test set, never both. The study employed **five-fold cross-validation** to evaluate the performance of the proposed time-distributed AlexNet model on the EduNet dataset. This method splits the dataset into five equal parts, using four parts for training and one part for testing in each iteration. The process was repeated over **10 runs** to ensure robustness and to account for variability in results.
- **Training–Testing Split:** For initial evaluations, the dataset was split into **80% training data** and **20% testing data**, ensuring a consistent and balanced approach to assess generalisation.
- **Evaluation Metrics Used:** We employed several metrics to assess the model’s performance, including overall accuracy, precision, recall, F1 score and the confusion matrix.

### 4.2. Experimental Results Without Noise Injection

In the beginning, the evaluation of the time-distributed AlexNet model applied to the EduNet dataset without any application of noise, the classification performance across various classroom activity classes yields insightful results. The performance metrics with the highest accuracy are shown in Table 5. These metrics indicate a high level of efficacy in recognising and distinguishing between the ten specified classroom behaviours. In our experiment, we divided the dataset into training and testing sets as 80% and 20%, respectively, where we had 743 training samples and 186 testing samples. The training configuration for the model employed the Adam optimiser due to its adaptive learning rate capabilities, which are essential for achieving faster convergence in deep learning networks. The model utilised a categorical cross-entropy loss function suitable for multi-class classification tasks. It was trained with a batch size of 8, carefully chosen to balance the trade-off between memory consumption and effective model performance. Furthermore, the training process was designed to continue for up to 100 epochs, incorporating an early stopping mechanism triggered by validation loss improvements to prevent over-training and ensure optimal generalisation.

To ensure the robustness of our evaluation, we performed five-fold cross-validation [52,53], repeating this process for 10 runs. The average performance and standard deviation [54] across these runs were calculated (see Table 6), providing a comprehensive assessment of the model’s consistency. The average overall accuracy achieved was 90.11% with a standard deviation of 0.62%.

**Figure 3:**
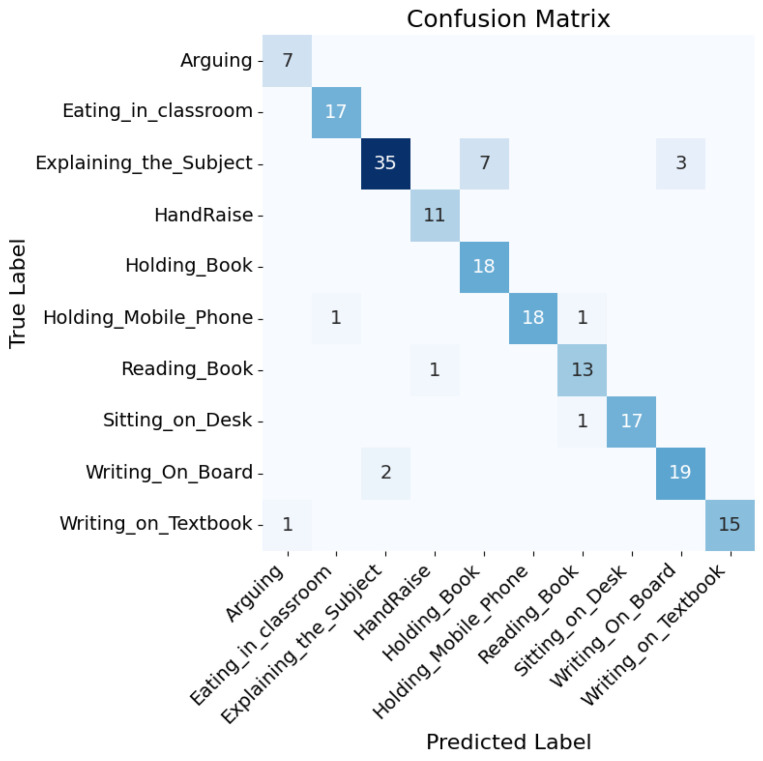
Heatmap of the confusion matrix.

The confusion matrix [55] related to the Table 5 shown in Figure 3 computed on the test set (20% split of EduNet dataset) offers insights into the model’s performance across various classes. The activity recognition performance of the model demonstrates varying degrees of accuracy across different activities. The activity of arguing was flawlessly identified in all seven instances without any misclassifications. The eating_in_classroom activity was mostly accurate, with 17 instances correctly classified, although there was one case where it was confused with holding_mobile_phone. Explaining_the_subject showed a high level of accuracy with 35 correct recognitions; however, there were some confusions leading to seven misclassifications with holding_book and three with writing_on_board. HandRaise was correctly identified in all 11 instances and holding_book was also perfectly recognised in all 18 cases without any errors. Holding_mobile_phone was mostly accurate with 18 correct classifications, but one instance was mistaken for eating_in_classroom. Reading_book was well classified with 13 correct recognitions, though one case was erroneously identified as HandRaise. Sitting_on_desk was correctly identified in 17 occurrences, with one misclassification as reading_book. Writing_on_board exhibited high accuracy with 19 correct recognitions, though two instances were misclassified as explaining_the_subject. Finally, writing_on_textbook was generally well classified with 15 correct recognitions, but there was one misclassification with arguing, indicating some visual similarities between these activities.

The model’s performance on the EduNet dataset, as detailed in the confusion matrix, showcases its capability to accurately identify classroom activities with varying degrees of complexity. Activities like “Arguing,” “HandRaise,” and “Holding_Book” were perfectly recognised without any misclassifications, indicating strong model reliability for distinct actions. However, some other activities involving more subtle differences in visual cues, such as “Explaining the Subject” and “Writing_on_board,” faced higher instances of misclassification due to their similarity to other action classes. This suggests that while the model is highly effective in distinguishing clear-cut activities, it encounters challenges when dealing with actions that share visual similarities.

The obtained results indicate the model’s robustness, particularly in distinguishing activities that have distinct visual cues. However, the errors in classifying actions with similar postures or hand movements point to potential areas for model refinement. Enhancing the model could involve more granular feature extraction techniques or incorporating additional contextual information to better differentiate between visually similar activities. The quality of the results is promising for the application of this model in educational tools, potentially aiding in the automated analysis of classroom dynamics to support pedagogical assessments and interventions.

#### 4.2.1. Comparison with Existing Approaches

In the domain of educational human activity recognition, the proposed time-distributed AlexNet model on the EduNet dataset is compared with two other methods, which were evaluated on the same dataset (see Table 7). We implemented ViT (Vision Transformer) [50], which achieved an accuracy of 76.88% and ConvLSTM model [51], which integrates convolutional layers with recurrent units, achieving an accuracy of 77.81%. However, it underperformed compared to our method, likely due to limitations in explicitly modelling frame-level independence and cross-frame consistency.

To further validate the observed performance differences, we conducted statistical comparisons. A paired *t*-test between the time-distributed AlexNet and ConvLSTM yielded a t-value of 31.82 and a *p*-value of $2.81\times 10^{-17}$, indicating a statistically significant improvement. Similarly, a paired *t*-test between the time-distributed AlexNet and ViT model resulted in a t-value of 55.13 and a *p*-value of $1.58\times 10^{-21}$. These results confirm that the proposed model offers a more accurate and stable solution for HAR in educational environments.

As shown in Table 7, the proposed time-distributed AlexNet contains substantially more parameters (58.36M) compared to ConvLSTM (7.49M) and ViT (1.84M). The performance advantage of the proposed time-distributed AlexNet over ConvLSTM and ViT can be attributed primarily to its higher parameter count and representational capacity. The additional integration of Gaussian noise serves as a complementary regularisation strategy, which provides incremental gains in generalisation and training stability, as shown in Section 4.2.2.

#### 4.2.2. Gaussian Noise Injection into Time-Distributed AlexNet

To assess the effect of Gaussian noise injection on training dynamics and classification accuracy, we compared two configurations of the time-distributed AlexNet model: one without noise (baseline) and another with Gaussian noise (standard deviation σ=0.01) applied at the input layer. Using five-fold cross-validation, the baseline model achieved an average accuracy of 90.11%, while the model trained with Gaussian noise injection achieved a higher average accuracy of 91.09%. This improvement indicates that noise injection serves as an effective regularisation strategy.

**Figure 4:**
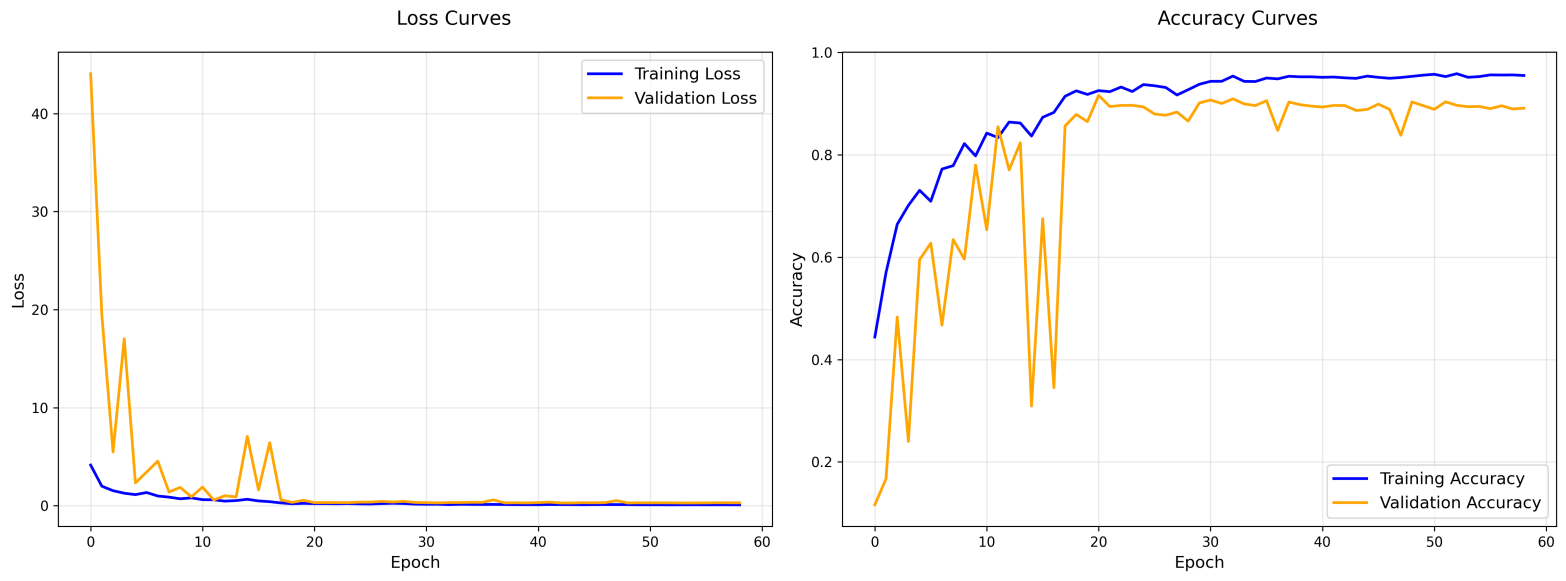
Loss and accuracy curves: training and validation without noise.

**Figure 5:**
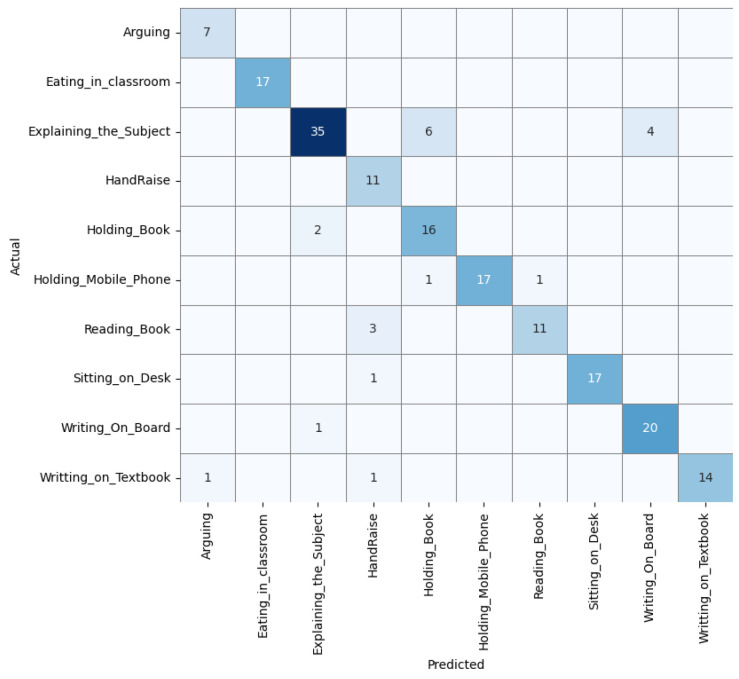
Confusion matrix without Gaussian noise.

The training and validation performance of a representative fold for the baseline model is shown in Figure 4. The training loss steadily decreased and training accuracy improved, but the validation accuracy exhibited fluctuations during early epochs, indicating potential overfitting or sensitivity to intra-class variations. The confusion matrix (Figure 5) further highlights notable misclassifications, particularly in classes such as *Explaining_the_Subject*, *Holding_Book* and *Reading_Book*.

**Figure 6:**
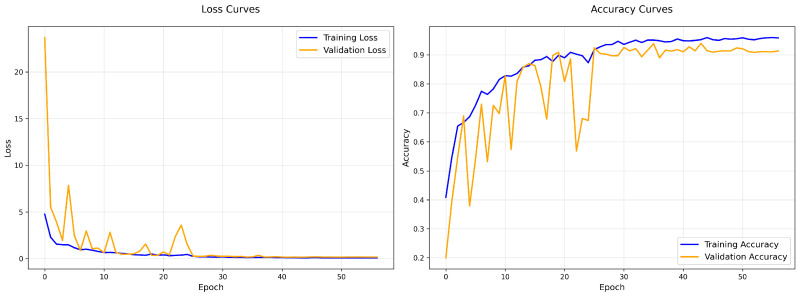
Loss and accuracy curves: training and validation with Gaussian noise (σ=0.01).

**Figure 7:**
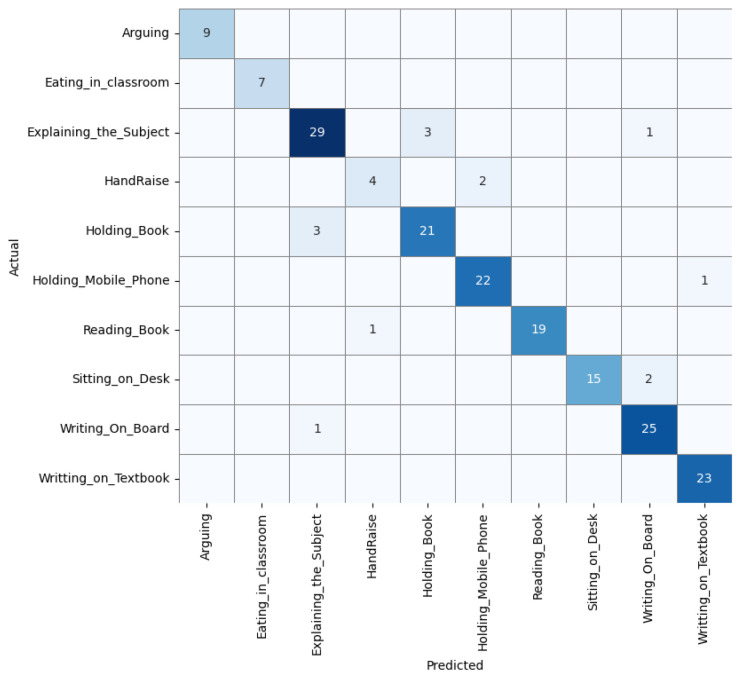
Confusion matrix with Gaussian noise (σ=0.01).

In contrast, the training curves for the noise-injected model (Figure 6) show better alignment between training and validation accuracy, with reduced variance and improved stability across epochs. The confusion matrix (Figure 7) reveals improved classification across most classes, although some confusion remains between visually similar actions like *Explaining_the_Subject*.

Overall, these results demonstrate that injecting mild Gaussian noise (σ=0.01) improves model generalisation and stabilises learning dynamics in video-based HAR, particularly in the presence of subtle inter-class visual differences.

Furthermore, the grid search [56] was performed over the range of Gaussian noise values (0.01, 0.02, 0.03, 0.04, 0.05, 0.06, 0.07, 0.08, 0.09, 0.1, 0.5, 1), providing a comprehensive view of the relationship between noise injection and model performance. The results indicated that Gaussian noise injection could improve the model’s ability to generalise, but the effect was highly dependent on the noise value chosen.

The noise value of 0.1 achieved the highest test accuracy in this study. However, performance was not entirely consistent across all runs, suggesting that while this noise level is optimal for generalisation, there is still some sensitivity to random initialisation and other factors.

As noise values increased, there was a noticeable decline in model performance. For instance, noise values such as 0.5 and 1.0 resulted in much lower accuracies, indicating that excessive noise can obscure the underlying patterns in the data to a point where the model can no longer generalise effectively.

This variability across different noise levels emphasises the importance of careful experimentation and tuning of the noise parameter. While noise injection can improve robustness, improper tuning can degrade performance, as observed with higher noise levels.

#### 4.2.3. Analysis of Gaussian Noise Injection and Its Effect

This section presents a detailed analysis of the effect of Gaussian noise injection on the model’s performance. The model was trained using 12 different Gaussian noise values (ranging from 0.01 to 1.0) and was evaluated across five different runs to measure the variability in test accuracy. The focus of this analysis is on the overall performance of the model, particularly highlighting the noise value of 0.1, which consistently performed well across different runs.

Table 8 presents the test accuracy for each noise value across the five experimental runs. The noise value of 0.1 consistently provided high accuracy, while higher noise values (e.g., 1.0) led to degradation in performance.

The impact of Gaussian noise injection on the HAR model’s performance, as displayed in Table 8, reveals several critical insights into how varying levels of noise influence test accuracy. By examining the performance across different noise values (ranging from 0.005 to 1.0), we can observe a clear relationship between noise level and the model’s ability to generalise effectively.

**Low Noise Values (0.005 to 0.01):** For the lowest noise values (0.005 to 0.01), the model achieved relatively high accuracy across all five runs. For instance, the noise value of 0.005 resulted in average test accuracies ranging from 83.40% to 89.08%, indicating that a minimal amount of noise injection helps prevent the model from overfitting to the training data. This moderate improvement suggests that adding a slight level of randomness to the input data enables the model to learn more generalised patterns rather than memorising specific details.

The noise value of 0.01 consistently achieved the highest test accuracy with an average accuracy of approximately 92.54% across all runs. This indicates that at very low noise levels, the model benefits from noise injection, enhancing its ability to handle variations in the data without being overwhelmed by the added randomness.

**Moderate Noise Values (0.02 to 0.1):** As the noise level increases to moderate values (0.02 to 0.1), there is a noticeable fluctuation in model performance. For example, with a noise value of 0.02, the average accuracy dropped to around 85.69%, which is lower than that of the 0.01 noise value. Similarly, at noise values such as 0.03, 0.04 and 0.05, the model’s accuracy remained relatively high but did not exceed the peak performance observed at the 0.01 level.

Interestingly, the noise value of 0.1 stood out as one of the most effective, achieving an average accuracy of 91.40%. This suggests that a noise value of 0.1 strikes a balance between adding enough randomness to enhance generalisation while not excessively distorting the input data. As seen from the results, this noise level allows the model to avoid overfitting while still capturing essential features required for accurate classification.

These observations imply that there is a threshold for noise injection where the positive effects of regularisation are maximised. Beyond this threshold, the benefits of noise injection start to diminish and the model’s performance begins to degrade.

**High Noise Values (0.5 and 1.0):** The most significant drop in performance was observed at the highest noise levels (0.5 and 1.0). With an average test accuracy of around 44.92% for a noise value of 0.5 and 22.19% for a noise value of 1.0, it is evident that the model struggled to extract meaningful patterns from the highly distorted data. At these levels, the injected noise overwhelmed the input features, making it difficult for the model to distinguish between relevant information and random variations.

This outcome highlights a critical drawback of excessive noise injection: while noise can help prevent overfitting, too much noise introduces substantial uncertainty, effectively “drowning out” the signal needed for accurate classification. The model’s inability to learn from such heavily corrupted data results in a reduction in performance, as observed with the lowest accuracy scores.

#### 4.2.4. Key Takeaways from the Noise Analysis

- **Optimal Noise Range:** The data indicates that the optimal noise level for this HAR model lies between 0.01 and 0.1. Within this range, the model consistently achieved high accuracy, with 0.1 emerging as the most effective noise value in balancing generalisation and performance.
- **Diminishing Returns with Increasing Noise:** As the noise level increases beyond the moderate range, the accuracy of the model begins to decline. This suggests that although noise injection is a valuable regularisation technique, there is a point where its benefits plateau and further increases in noise become detrimental to performance.
- **Negative Impact of High Noise Values:** Excessive noise (values of 0.5 and 1.0) has a clearly adverse effect on the model’s performance. These high noise levels introduce too much randomness, leading to a dramatic loss of accuracy and the model’s inability to learn effectively from the input data.

The detailed analysis from Table 8 demonstrates that Gaussian noise injection can be a powerful tool for improving model generalisation when applied correctly. The key to leveraging noise injection lies in selecting an appropriate noise level, as shown by the superior performance at noise values around 0.01 to 0.1. However, when noise injection is not carefully tuned, it can hinder model performance, as seen with the accuracy drop at higher noise values.

#### 4.2.5. Statistical Analysis

To rigorously evaluate the effect of Gaussian noise injection on model performance, we conducted a statistical comparison between the baseline model and the model incorporating a Gaussian noise value of 0.01. Both models were trained using five-fold cross-validation and each experiment was repeated 10 times to ensure the reliability of the results (see Table 9).

- Statistical Comparison:
  To determine whether the observed performance difference between the two models was statistically significant, we conducted a two-sample *t*-test on the sets of accuracy values obtained across the 10 runs. The test produced a t-value of −2.83 and a corresponding *p*-value of 0.01106.
  This result indicates statistical significance at the commonly accepted α=0.05 level, allowing us to reject the null hypothesis that there is no difference in performance between the models. The improvement in accuracy with noise injection is therefore unlikely to have occurred by chance.
- Interpretation:
  The statistical analysis confirms that injecting Gaussian noise with σ=0.01 results in a meaningful and consistent improvement in model performance. The lower standard deviation also indicates more stable convergence. These findings support the effectiveness of noise injection as a regularisation strategy to enhance generalisation in recognition tasks using the EduNet dataset.

#### 4.2.6. Impact of Learning Rate on Model Performance

Learning rate [57,58] plays a pivotal role in the performance and stability of deep learning models, directly influencing convergence and generalisation. To explore this relationship, statistical *t*-tests [59] were conducted to evaluate the impact of various learning rates on the performance of the time-distributed AlexNet model. The learning rate of 0.0001 is used as a reference point because it represents a widely accepted baseline that balances stability and convergence, providing a reliable benchmark for assessing the impact of varying learning rates on the model’s performance. Its performance was compared against other learning rates ranging from 0.00005 to 0.001. The statistical evaluation is summarised in the Table 10.

#### 4.2.7. Key Observations

- **Significant Differences at Certain Learning Rates:** Statistically significant differences in performance were observed between the learning rate of 0.0001 and the learning rates of 0.0006, 0.0007 and 0.0008, as indicated by their *p*-values [59] (Table 10).
- **No Significant Differences Across Most Learning Rates:** For smaller learning rates, such as 0.00005, 0.00006 and 0.00007, no significant differences in performance were observed.
- **Importance of Fine-Grained Tuning:** While 0.0001 provided a robust baseline, the evaluation highlights the importance of fine-grained learning rate adjustments. Intermediate rates, such as 0.0007, offered a unique balance of high accuracy and stability, making them promising candidates for further exploration.

The analysis underscores the critical impact of learning rate selection on the time-distributed AlexNet model’s performance for human activity recognition tasks. Smaller learning rates exhibited improved stability, with notable reductions in variability across experimental runs. While the baseline rate of 0.0001 provided a strong foundation, the potential of intermediate rates, such as 0.0006 to 0.0008, for enhancing performance warrants further investigation.

These findings emphasise the importance of optimising learning rates to balance convergence, generalisation and stability. The insights gained from this study contribute to the broader understanding of hyperparameter tuning and its role in advancing deep learning models for real-world applications.

#### 4.2.8. Comparison with Traditional Regularisation Techniques

To further evaluate the effectiveness of Gaussian noise injection, we compared it against traditional L2 regularisation. Both models included standard regularisation components—such as Batch Normalisation and Dropout—as part of the base time-distributed AlexNet architecture. We trained the model using L2 regularisation and performed five-fold cross-validation, achieving an average accuracy of 80.73%, precision of 85.71%, recall of 80.73% band F1 score of 81.12%. In contrast, Gaussian noise injection with σ=0.01 yielded a higher average accuracy of 92.54%, as detailed in Table 8. While L2 regularisation penalises large weights to reduce overfitting, Gaussian noise injection perturbs the input distribution, encouraging the model to learn more robust and invariant spatiotemporal features. Given that both techniques were evaluated within an identical architectural and training setup, these findings indicate that biologically inspired noise injection offers superior generalisation, particularly for activity recognition in egocentric classroom video data, where environmental variability is prominent.

### 4.3. Computational Cost Analysis

Here, we evaluate computational efficiency and we provide a comparative analysis of the computational cost for the proposed and baseline models, both with and without noise injection, alongside alternative methods. The analysis includes Floating-Point Operations per second (FLOPs) and the number of trainable parameters. FLOPs were estimated using the keras-flops tool with a batch size of 1 and input shape of (25,224,224,3). All models were implemented and profiled under equivalent input and training configurations to ensure fair comparison.

To evaluate the computational efficiency of the proposed and baseline models, we computed both the number of Floating-Point Operations (FLOPs) and trainable parameters using the keras-flops tool. The analysis was conducted using a batch size of 1 and an input resolution of (25,224,224,3), corresponding to 25-frame video clips.

As shown in Table 11, the ConvLSTM model achieves a good balance between computational cost and capacity with 5.26 GFLOPs and 7.49 million parameters. The time-distributed AlexNet-based models (with and without Gaussian noise) are significantly heavier, requiring 56.83 GFLOPs and 58.36 million parameters. On the other hand, the ViT transformer-based model is surprisingly lightweight, with only 2.72 GFLOPs and 1.84 million parameters, making it a computationally efficient alternative.

It is important to note that the FLOPs reported in Table 11 represent inference-only complexity as computed using the keras-flops tool. Since the GaussianNoise layer is non-trainable and only active during training [60], its computational cost is not reflected in these values. Specifically, the GaussianNoise layer does not contain any learnable parameters; it simply adds random perturbations sampled from a Gaussian distribution $N(0,\sigma^{2})$ to the input frames during training. The noise level σ is a fixed hyperparameter defined by the user and the layer is automatically bypassed during inference to ensure deterministic predictions. While this introduces a negligible overhead during training due to element-wise noise addition, the effect is minimal compared to the convolutional and fully connected operations that dominate computational complexity. Most importantly, Gaussian noise injection does not alter the model’s architecture or inference-time efficiency, meaning that FLOPs and parameter counts remain identical for both the baseline and noise-injected variants. This ensures that the method delivers measurable improvements in generalisation without incurring additional deployment costs.

### 4.4. Analysis of the Model Performance on UCF50 and UCF101 Datasets with Gaussian Noise Injection

While the primary focus was the evaluation on the EduNet dataset, we conducted further experiments on the UCF50 and UCF101 datasets to assess whether the effects of Gaussian noise observed on EduNet generalise to broader action recognition tasks. Initially, we experimented without noise (0.000) and then the noise was applied to the input video frames with standard deviations ranging from 0.005 to 1.000 across five independent runs. The objective was to examine the robustness and generalisation ability of the proposed time-distributed AlexNet model under varying noise perturbations.

As shown in Table 12, a clear trend emerges: introducing low to moderate levels of Gaussian noise (σ=0.02 to 0.04) consistently improves or maintains classification performance, whereas excessive noise (σ=0.5 and above) severely degrades accuracy.

The baseline model without any noise injection (σ=0.000) achieved an average accuracy of **87.88%**. In comparison, the highest accuracy was recorded at σ=0.04, reaching **91.45%**, followed by σ=0.02 with **90.93%**. This improvement suggests that mild stochastic perturbations during training act as effective regularisers, reducing overfitting and enhancing generalisation. These findings align with established theories in deep learning, where noise helps smooth decision boundaries and foster more robust feature representations.

Conversely, the model’s performance deteriorated under high noise conditions: σ=0.5 led to an average accuracy of only **49.33%** and further dropped to **10.15%** at σ=1.0. Such excessive noise disrupts the discriminative spatial–temporal patterns required for accurate human activity recognition.

Compared to results on the EduNet dataset, similar conclusions hold—moderate noise enhances performance, while too much noise is detrimental. However, UCF50 showed more robustness across a wider noise range, possibly due to its more constrained and visually consistent action categories.

In conclusion, this evaluation reinforces the effectiveness of Gaussian noise injection as a lightweight regularisation technique. Based on the UCF50 results, the optimal performance is achieved with a noise standard deviation between **0.02 and 0.04**, which provides the best balance between robustness and accuracy when compared to the noise-free baseline.

On the other hand, we conducted another experiment on the UCF101 dataset. Gaussian noise with varying standard deviations—from 0.000 (no noise) to 1.000—was introduced during training and each configuration was evaluated across five independent runs. Table 13 presents the detailed results.

The baseline model, trained without any noise (σ=0.000), achieved the highest average accuracy of **88.66%**. Interestingly, low levels of Gaussian noise between σ=0.005 and 0.020 produced comparable results, with average accuracies ranging from **88.30%** to **88.58%**. The highest accuracy among noisy models was observed at σ=0.009 with an average of **88.58%**, indicating that minor stochastic perturbations can act as a soft regulariser without degrading performance.

However, as the noise level increased beyond σ=0.050, a sharp decline in accuracy was observed. For example, the accuracy dropped to **86.49%** at σ=0.050 and further plummeted to **29.57%** and **1.95%** at σ=0.500 and σ=1.000, respectively. These results reaffirm that while mild noise levels may be tolerated or even beneficial, excessive noise can obscure the informative signal and severely hamper learning.

Overall, the analysis confirms that while Gaussian noise injection offers a lightweight regularisation technique, its benefits are bounded. Interestingly, for the large-scale UCF101 dataset, noise injection slightly reduced performance compared to the baseline model. This can be attributed to the inherent richness and diversity of UCF101, which already promotes generalisation. In such cases, additional noise may introduce excessive perturbations, thereby diminishing accuracy. This suggests that the effectiveness of noise injection is dataset-dependent and most beneficial in smaller or more constrained scenarios, such as EduNet.

## 5. Discussion

The results from the time-distributed AlexNet model tested on the EduNet dataset showed a nuanced understanding of classroom dynamics through video data. The model demonstrates high efficacy in recognising specific classroom behaviours. These metrics indicate robust performance across varied educational activities, aligning with the objectives of enhancing automated classroom monitoring tools.

### 5.1. Practical Implications

The application of this model can impact educational technology, particularly in developing intelligent monitoring systems that support both teachers and students in various educational contexts [61], such as the following.

- **Enhanced Learning Environments:** Automated feedback on classroom engagement and student behaviour could help educators adjust their teaching strategies in real time. This dynamic adaptation can potentially lead to improved educational outcomes and more personalised learning experiences.
- **Focused Educational Interventions:** Identification of specific behaviours like ‘HandRaise’ or ‘Holding_Mobile_Phone’ allows for tailored interventions to improve student attentiveness or manage classroom discipline. Such targeted strategies can enhance the effectiveness of educational efforts.
- **Educational Content Analysis:** The technology could be employed in online learning platforms to assess the effectiveness of educational content based on student interaction and engagement. This application can help refine online educational materials and methods, ensuring they meet the diverse needs of learners [62].

### 5.2. Challenges and Limitations

The study encountered some challenges and has some limitations, which are:

- **Over-Classification and Imbalance:** The discrepancy in the confusion matrix, where some classes were predicted more frequently than others, highlights potential issues with the class imbalance and the model’s tendency to over-classify certain behaviours. For instance, despite having the most samples (186), Explaining_the _subject demonstrated a high degree of accuracy with 35 correct recognitions; however, there were some misunderstandings that resulted in seven misclassifications with holding_a_book and three with writing_on_a_board. This could be mitigated by implementing more balanced dataset preparation or advanced sampling techniques [63].
- **Complexity of Human Behaviour:** The model’s difficulty in accurately distinguishing between similar activities like ‘Explaining_the_Subject’ and ‘Holding_Book’ underscores the complexity of human behaviour and the limitations of current feature extraction methods in CNNs [64]. Future work could explore deeper, more complex network architectures.
- **Generalisation Across Different Environments:** The current dataset, while robust, may not adequately represent the global diversity of classroom activities. The model’s ability to generalise across different cultural or educational settings remains a question and necessitates further validation [65].

Moving forward, addressing these challenges will require a focus on refining data collection processes, enhancing model architectures and continually testing the system’s applicability in diverse educational contexts. Additionally, ethical considerations around privacy and consent in real-world applications must be rigorously maintained to ensure that such technologies are used responsibly and beneficially within educational settings.

### 5.3. Limitations and Future Work

The findings from this research underscore the potential of noise-injected deep learning models for robust performance in complex scenarios. However, several limitations and avenues for future development remain. One of the key challenges lies in the fine-tuning of noise injection parameters, including amplitude and type, which is both computationally intensive and highly dataset-dependent [66]. Future work could explore dynamic noise profiles, where noise levels adapt according to node activation levels or learning phases. This adaptive approach may reduce unnecessary perturbations and further enhance learning dynamics [67].

Incorporating alternative noise types, such as Laplacian [68] or chaotic noise [69] could provide additional regularisation benefits. For example, chaotic noise may help prevent premature convergence, especially in datasets with complex spatiotemporal dependencies. Furthermore, hybrid strategies that combine noise injection with existing regularisation techniques, such as dropout [46] or Bayesian approximations [70], could further improve the robustness and generalisation of the model.

Looking ahead, the integration of multimodal architectures capable of capturing the nuanced nature of human activity presents a compelling research direction [71]. By incorporating additional data modalities—such as audio, textual cues, or physiological signals—models can achieve a more holistic understanding of behavior [72,73]. The combination of such multimodal inputs with noise-augmented learning mechanisms may result in even more resilient activity recognition systems [74].

Another important limitation of this study is that the evaluation on the EduNet dataset was conducted on a subset (10 classes) of the complete corpus. While this subset provided sufficient diversity to demonstrate the effectiveness of the proposed noise-injected time-distributed AlexNet, it may not fully capture the variability present in the entire dataset. Future work will extend the evaluation to the full EduNet dataset with 20 classes as well as additional large-scale benchmarks (e.g., Kinetics) to provide a more comprehensive assessment of robustness and scalability.

Validation across a broader spectrum of datasets and contexts—including different educational settings and cultural environments—remains essential for assessing the true generalisability of the proposed approach [75]. While preliminary results are promising, scaling this method to real-world deployments introduces several important considerations. The model’s architecture, which leverages a time-distributed AlexNet backbone, imposes substantial computational demands, particularly when processing sequences of high-resolution frames. Although feasible within a high-performance computing environment, such requirements may hinder deployment on resource-limited devices or in real-time systems. Techniques such as model compression, pruning and quantisation should be explored to mitigate these constraints.

Furthermore, adapting the model to domains beyond classroom activity recognition may require addressing issues such as domain shift, label imbalance and variations in activity granularity. Transfer learning and domain adaptation strategies could enhance the model’s scalability and performance across different application areas.

Finally, ethical considerations, including privacy, surveillance implications and informed consent, must be central to the development and deployment of noise-injected HAR systems. Future work should contribute to the establishment of robust guidelines and responsible AI frameworks tailored to activity recognition in sensitive environments [70].

By addressing these technical, ethical and operational challenges, future research can advance the applicability, adaptability and responsible deployment of noise-injected neural network models for HAR.

## 6. Conclusions

This research presented a comprehensive investigation into the use of deep learning techniques for human activity recognition (HAR) in educational environments, specifically focusing on classroom activities using the EduNet dataset. The research demonstrated the successful implementation of a time-distributed AlexNet architecture, achieving a high classification accuracy of 90.11%. This performance highlights the model’s effectiveness in identifying specific classroom behaviours, contributing to the development of automated classroom monitoring systems. The ability to classify complex activities such as hand-raising, reading and arguing through video data marks a valuable step toward advancing automated educational tools.

However, the analysis revealed certain challenges related to class balance and model training. Discrepancies in the confusion matrix showed that some activities were over-represented while others were under-represented, indicating potential areas for improvement in dataset management and model training methodologies. Addressing these issues will be crucial to refining the system’s accuracy and ensuring it provides reliable outputs across all classes.

Additionally, this research explored the application of Gaussian noise injection as a regularisation technique. The results confirmed that moderate noise levels, particularly around a noise value of 0.01, enhanced the model’s generalisation abilities by preventing overfitting and allowing the network to capture meaningful patterns from the data. Conversely, excessive noise injection proved detrimental, reducing the model’s ability to extract valuable features due to the overly distorted input data. This highlights the importance of carefully tuning noise levels to strike a balance between variability and data integrity.

The findings from this research demonstrate that noise injection can be an effective tool for improving model robustness, especially when combined with other regularisation techniques like Batch Normalisation and Dropout. However, careful experimentation is essential to ensure optimal performance. These insights contribute to the broader discourse on the use of machine learning technologies in educational research, showing how they can facilitate the automated analysis of student engagement and classroom interaction.

In conclusion, the successful application of deep learning to HAR, combined with insights into noise injection, represents a promising avenue for future research in automated educational systems. Continued exploration of adaptive noise injection strategies and refined training methodologies will further enhance the effectiveness of these systems in real-world educational environments.

## Figures and Tables

**Table 2:** Time-distributed AlexNet: architecture details.

Component	Description
Input	Sequence of 25 RGB frames: (25, 224, 224, 3)
Conv1	TimeDistributed Conv2D: 96 filters, 11 × 11 kernel, stride 4 → (25, 56, 56, 96)
MaxPool1	TimeDistributed MaxPooling2D: 3 × 3, stride 2 → (25, 27, 27, 96)
Conv2	TimeDistributed Conv2D: 256 filters, 5 × 5 kernel → (25, 27, 27, 256)
MaxPool2	TimeDistributed MaxPooling2D: 3 × 3, stride 2 → (25, 13, 13, 256)
Conv3	TimeDistributed Conv2D: 384 filters, 3 × 3 kernel → (25, 13, 13, 384)
Conv4	TimeDistributed Conv2D: 384 filters, 3 × 3 kernel → (25, 13, 13, 384)
Conv5	TimeDistributed Conv2D: 256 filters, 3 × 3 kernel → (25, 13, 13, 256)
MaxPool3	TimeDistributed MaxPooling2D: 3 × 3, stride 2 → (25, 6, 6, 256)
Flatten	TimeDistributed(Flatten) → (25, 9216)
Concatenation	Keras-implicit flattening of (25, 9216) → (230400)
Dense1	Dense Layer: 4096 units, ReLU
Dense2	Dense Layer: 4096 units, ReLU
Output	Dense Layer: 10 units, softmax

**Table 3:** Distribution of samples across different classes in the EduNet dataset.

Class	No. of Samples	Student/Teacher Centric Cases
Arguing	64	Student
Eating in Classroom	60	Student
Explaining the Subject	186	Teacher
Hand Raise	53	Student
Holding Book	85	Teacher
Holding Mobile Phone	90	Teacher
Reading Book	91	Student
Sitting on Desk	72	Student
Writing on Board	133	Teacher
Writing on Textbook	95	Student

**Table 4:** Model hyperparameters—time-distributed AlexNet.

Hyperparameter	Value
Input Frame Size	224×224 pixels
Sequence Length (*T*)	25 frames
Batch Size	8
Optimiser	Adam
Learning Rate	0.0001 (tuned via grid search)
Loss Function	Categorical cross-entropy
Dropout Rate	0.5 (after fully connected layers)
Batch Normalisation	Applied after each convolutional layer
Gaussian Noise Injection	σ=0.01, applied at input layer
Epochs	Up to 100
Early Stopping Patience	15 epochs
Trainable Parameters	58,341,450
Non-trainable Parameters	19,136
Total Parameters	58,360,586

**Table 5:** Performance metrics.

Metric	Value (%)
Overall Accuracy	91.40
Precision	91.94
Recall	94.40
F1 Score	92.77

**Table 6:** Cross-validation metrics.

Metric	Value (%)
Average Accuracy	90.11
Average Precision	90.89
Average Recall	90.22
Average F1 Score	90.24

**Table 7:** Comparison of accuracy results and model complexity on the EduNet dataset.

Method	Accuracy (%)	Parameters (Millions)
ViT [50]	76.88	1.84
ConvLSTM [51]	77.81	7.49
Time-Distributed AlexNet (Ours)	90.11	58.36

**Table 8:** Test accuracies for different Gaussian noise values.

Noise Value	Run1 Accuracy (%)	Run2 Accuracy (%)	Run3 Accuracy (%)	Run4 Accuracy (%)	Run5 Accuracy (%)	Average Accuracy (%)
0.005	86.65	88.82	89.08	83.40	88.54	87.30
0.006	84.19	88.69	87.20	91.16	90.75	88.00
0.007	86.82	83.55	90.80	89.85	83.23	86.85
0.008	90.67	88.97	87.57	88.41	91.38	89.40
0.009	75.91	88.58	85.29	87.89	89.66	85.87
**0.01**	**93.23**	**92.14**	**92.56**	**92.78**	**91.97**	**92.54**
0.02	85.12	85.89	86.32	85.67	85.43	85.69
0.03	89.81	89.34	89.91	89.67	89.23	89.59
0.04	90.67	90.45	90.23	90.78	90.34	90.49
0.05	89.87	89.54	89.45	89.89	89.56	89.66
0.06	89.44	89.67	89.34	89.56	89.12	89.43
0.07	90.47	90.12	90.34	90.23	90.56	90.34
0.08	89.38	89.12	89.56	89.67	89.45	89.44
0.09	84.56	84.12	84.34	84.56	84.23	84.36
0.1	91.33	91.12	91.45	91.78	91.34	91.40
0.5	44.67	45.12	44.56	45.34	44.89	44.92
1.0	22.26	21.78	22.34	22.12	22.45	22.19

**Table 9:** Accuracy comparison across 10 runs: baseline vs. noise injection (σ=0.01).

Run	Baseline Accuracy (%)	Noise Injection Accuracy (%)
1	90.42	91.07
2	89.78	90.50
3	90.91	91.71
4	91.22	91.28
5	90.05	91.17
6	88.87	91.71
7	90.04	90.85
8	91.30	90.96
9	89.67	91.93
10	90.76	90.24
**Mean**	**90.30**	**91.14**
**Standard Deviation**	**0.77**	**0.54**

**Table 10:** Performance (in %) and standard deviations for different learning rates.

Run	0.00005	0.00006	0.00007	0.00008	0.00009	0.0002	0.0003	0.0004	0.0005	0.0006	0.0007	0.0008	0.0009	0.001	0.0001
1	91.51	90.32	90.32	90.52	90.43	89.61	92.99	88.97	89.91	86.09	83.10	88.65	88.80	86.67	91.05
2	88.45	92.13	89.18	91.27	89.66	92.09	88.45	91.53	89.72	89.68	86.75	90.10	90.97	90.99	90.90
3	91.68	89.63	90.60	89.01	90.60	86.80	91.63	81.57	91.31	88.04	88.00	86.77	91.81	89.70	89.33
4	92.22	91.78	91.96	89.03	92.47	91.94	92.97	88.13	90.69	86.28	87.59	88.02	90.62	89.89	91.70
5	91.25	90.43	92.28	91.89	88.28	90.49	92.13	92.17	87.13	90.95	87.81	89.79	86.82	91.20	91.32
**Std Dev.**	**1.48**	**1.05**	**1.27**	**1.30**	**1.53**	**2.16**	**1.87**	**4.21**	**1.60**	**2.12**	**2.04**	**1.35**	**2.00**	**1.80**	**0.91**
**Average**	**90.62**	**90.26**	**90.87**	**90.34**	**90.69**	**90.19**	**91.63**	**88.87**	**89.75**	**88.61**	**86.65**	**88.67**	**89.84**	**89.69**	**90.86**
***p*** **-Value (<0.05)**	**0.84**	**1.00**	**0.99**	**0.49**	**0.49**	**0.54**	**0.43**	**0.25**	**0.21**	**0.03**	**0.00**	**0.02**	**0.31**	**0.23**	**–**

**Table 11:** Computational cost comparison of video classification models.

Model	FLOPs (GFLOPs)	Trainable Parameters (Millions)
ConvLSTM	5.26	7.49
TD AlexNet	56.83	58.36
TD AlexNet + Gaussian Noise	56.83	58.36
ViT	2.72	1.84

**Table 12:** Test Accuracies for different Gaussian noise values on UCF50 dataset (5 runs).

Noise Value	Run1 Accuracy (%)	Run2 Accuracy (%)	Run3 Accuracy (%)	Run4 Accuracy (%)	Run5 Accuracy (%)	Average Accuracy (%)
0.000	88.07	88.12	88.47	88.15	86.66	87.88
0.005	90.73	90.81	90.65	90.85	90.60	90.73
0.006	90.65	90.72	90.57	90.77	90.52	90.65
0.007	90.05	90.18	89.97	90.17	89.92	90.06
0.008	90.20	90.35	90.12	90.32	90.07	90.21
0.009	90.43	90.50	90.35	90.55	90.30	90.43
0.010	89.68	89.75	89.60	89.80	89.55	89.68
0.020	90.95	91.02	90.87	90.70	91.10	90.93
0.030	90.73	90.80	90.65	90.48	90.88	90.71
**0.040**	**91.47**	**91.55**	**91.39**	**91.22**	**91.62**	**91.45**
0.050	90.28	90.35	90.20	89.50	90.43	90.15
0.060	87.36	87.43	87.28	86.58	88.00	87.33
0.070	89.23	89.30	89.15	88.45	89.87	89.20
0.080	90.05	90.12	89.97	89.27	90.69	90.02
0.090	87.58	87.65	87.50	86.80	88.22	87.55
0.100	89.90	89.97	89.82	89.12	90.54	89.87
0.500	49.89	49.95	49.83	45.00	52.00	49.33
1.000	10.25	10.30	10.20	9.00	11.00	10.15

**Table 13:** Test Accuracies for different Gaussian noise values on UCF101 dataset (5 runs).

Noise Value	Run1 Accuracy (%)	Run2 Accuracy (%)	Run3 Accuracy (%)	Run4 Accuracy (%)	Run5 Accuracy (%)	Average Accuracy (%)
**0.000**	**88.90**	**87.42**	**88.19**	**89.03**	**89.76**	**88.66**
0.005	88.62	88.39	88.46	88.70	88.07	88.45
0.006	88.24	88.99	88.92	88.40	88.32	88.57
0.007	87.40	88.67	88.61	87.38	88.86	88.18
0.008	88.18	88.11	88.66	88.76	88.02	88.34
0.009	88.56	88.41	88.34	88.64	88.93	88.58
0.010	88.12	88.66	88.34	88.40	88.70	88.44
0.020	88.73	87.97	88.51	87.79	88.48	88.30
0.030	88.15	87.68	87.94	87.49	88.03	87.86
0.040	87.41	88.23	88.05	87.58	87.54	87.76
0.050	86.57	86.08	86.65	87.04	86.12	86.49
0.060	86.51	86.54	86.42	86.89	86.76	86.62
0.070	85.94	85.02	85.97	86.15	85.31	85.68
0.080	84.76	84.45	85.61	85.93	85.85	85.32
0.090	85.86	84.80	84.62	84.88	84.69	84.97
0.100	84.51	85.94	85.18	84.50	82.60	84.55
0.500	24.60	32.52	27.63	35.04	28.04	29.57
1.000	1.88	2.99	1.37	1.41	2.11	1.95

## Data Availability

Data used in this work are publicly available. The source code is publicly available at the GitHub repository: https://github.com/sanjay-dutta/TimeDistributed-AlexNet (accessed on 30 June 2025).

## References

[1] Shin J., Hassan N., Miah A., Nishimura S. (2024). A comprehensive methodological survey of human activity recognition across diverse data modalities. arXiv.

[2] Djenouri Y., Belbachir A., Srivastava G., Cano A. (2024). Vision-based spatiotemporal learning for human activity recognition. Proceedings of the 2024 International Joint Conference on Neural Networks (IJCNN).

[3] Kidu T., Song Y., Seo K.W., Lee S., Park T. (2024). An intelligent real-time driver activity recognition system using spatio-temporal features. Appl. Sci..

[4] Wang H., Pu Z., Yi J., Qiu T., Liu Z., Liu Z. (2019). Time-sequence action-decision and navigation through stage deep reinforcement learning in complex dynamic environments. Proceedings of the 2019 IEEE Symposium Series on Computational Intelligence (SSCI).

[5] Krizhevsky A., Sutskever I., Hinton G.E., Pereira F., Burges C.J.C., Bottou L., Weinberger K.Q. (2012). ImageNet Classification with Deep Convolutional Neural Networks. Advances in Neural Information Processing Systems 25 (NeurIPS 2012).

[6] Zhang Z., Lv Z., Gan C., Zhu Q. (2020). Human action recognition using convolutional LSTM and fully-connected LSTM with different attentions. Neurocomputing.

[7] Nguyen H., Ribeiro B. (2023). Video action recognition collaborative learning with dynamics via PSO-ConvNet Transformer. Sci. Rep..

[8] Jegham I., Khalifa A., Alouani I., Mahjoub M. (2020). Vision-based human action recognition: An overview and real world challenges. Forensic Sci. Int. Digit. Investig..

[9] Anides E., Garcia L., Sanchez G., Avalos J.G., Abarca M., Frias T., Vazquez E., Juarez E., Trejo C., Hernandez D. (2022). A biologically inspired spiking neural P system in selective visual attention for efficient feature extraction from human motion. Front. Robot. AI.

[10] Santer R.D., Akanyeti O., Endler J.A., Galván I., Okal M.N. (2023). Why are biting flies attracted to blue objects?. Proc. R. Soc. B.

[11] Pratte M.S., Ling S., Swisher J.D., Tong F. (2013). How attention extracts objects from noise. J. Neurophysiol..

[12] Kukavčka J., Golkov V., Cremers D. (2017). Regularization for deep learning: A taxonomy. arXiv.

[13] Wikipedia Gaussian Noise. https://en.wikipedia.org/wiki/Gaussian_noise.

[14] Bishop C.M. (1995). Training with noise is equivalent to Tikhonov regularization. Neural Comput..

[15] Camuto A., Willetts M., Simsekli U., Roberts S.J., Holmes C.C. (2020). Explicit regularisation in gaussian noise injections. Adv. Neural Inf. Process. Syst..

[16] Duan F., Chapeau-Blondeau F., Abbott D. (2024). Optimized injection of noise in activation functions to improve generalization of neural networks. Chaos Solitons Fractals.

[17] Feng R., Zhao D., Zha Z.J. Understanding noise injection in gans. Proceedings of the International Conference on Machine Learning, PMLR.

[18] Ferianc M., Bohdal O., Hospedales T., Rodrigues M. (2023). Navigating noise: A study of how noise influences generalisation and calibration of neural networks. arXiv.

[19] Zhou J., Ran F., Li G., Peng J., Li K., Wang Z. (2022). Classroom learning status assessment based on deep learning. Math. Probl. Eng..

[20] Pang S., Lai S., Zhang A., Yang Y., Sun D. (2023). Graph convolutional network for automatic detection of teachers’ nonverbal behavior. Comput. Educ. Artif. Intell..

[21] Hua Y., Todo Y., Tao S., Tang Z., Cheng T., Qiu Z. (2024). Bio-inspired computational model for direction and speed detection. Knowl.-Based Syst..

[22] Lalwani P., Ramasamy G. (2024). Human activity recognition using a multi-branched CNN-BiLSTM-BiGRU model. Appl. Soft Comput..

[23] Benedetti M., Ventura E. (2024). Training neural networks with structured noise improves classification and generalization. J. Phys. A Math. Theor..

[24] Athilakshmi R., Varsha S., Hirani U., Salomi M., Meenakshi N. (2024). Spatio-Temporal Fusion for Video-Based Human Activity Recognition: A Novel LRCN TDL Approach. Proceedings of the 2024 International Conference on Data Science and Network Security (ICDSNS).

[25] Sharma V., Gupta M., Kumar A., Mishra D. (2024). STAR-3D: A holistic approach for human activity recognition in the classroom environment. Information.

[26] Zheng R., Jiang F., Shen R. Intelligent student behavior analysis system for real classrooms. Proceedings of the IEEE International Conference on Acoustics, Speech and Signal Processing (ICASSP).

[27] Pervaiz M., Akhter I., Chelloug S. (2022). An optimized system for human behaviour analysis in E-learning. Proceedings of the 2022 International Conference on Electrical Engineering and Sustainable Technologies (ICEEST).

[28] Herrmann B. (2025). Enhanced neural speech tracking through noise indicates stochastic resonance in humans. eLife.

[29] Kim Y., Soyata T., Behnagh R. (2018). Towards emotionally aware AI smart classroom: Current issues and directions for engineering and education. IEEE Access.

[30] Nida N., Yousaf M., Irtaza A., Velastin S. (2019). Instructor activity recognition through deep spatiotemporal features and feedforward extreme learning machines. Math. Probl. Eng..

[31] Gupta S., Ashwin T., Guddeti R. (2019). Students’ affective content analysis in smart classroom environment using deep learning techniques. Multimed. Tools Appl..

[32] Behera A., Matthew P., Keidel A., Vangorp P., Fang H., Canning S. (2020). Associating facial expressions and upper-body gestures with learning tasks for enhancing intelligent tutoring systems. Int. J. Artif. Intell. Educ..

[33] Cheng Y., Dai Z., Ji Y., Li S., Jia Z., Hirota K., Dai Y. Student action recognition based on deep convolutional generative adversarial network. Proceedings of the Chinese Control and Decision Conference (CCDC).

[34] Tonguç G., Ozkara B. (2020). Automatic recognition of student emotions from facial expressions during a lecture. Comput. Educ..

[35] Nguyen D., Nguyen X., Than T., Nguyen M. Automated attendance system in the classroom using artificial intelligence and internet of things technology. Proceedings of the 8th NAFOSTED Conference on Information and Computer Science (NICS).

[36] Gang Z., Wenjuan Z., Biling H., Jie C., Hui H., Qing X. (2021). A simple teacher behavior recognition method for massive teaching videos based on teacher set. Appl. Intell..

[37] Sharma V., Gupta M., Kumar A., Mishra D. (2021). EduNet: A New Video Dataset for Understanding Human Activity in the Classroom Environment. Sensors.

[38] Su K., Li X., Zhou C., Chen X. (2021). Learning behaviour recognition based on multi-object image in single viewpoint. Pers. Ubiquitous Comput..

[39] Lin F.C., Ngo H.H., Dow C.R., Lam K.H., Le H. (2021). Student behavior recognition system for the classroom environment based on skeleton pose estimation and person detection. Sensors.

[40] Rafique M., Khaskheli F., Hassan M., Naseer S., Jeon M. (2022). Employing automatic content recognition for teaching methodology analysis in classroom videos. PLoS ONE.

[41] Qiu Q., Wang T., Chen F., Wang C. (2023). LD-Recognition: Classroom action recognition based on passive RFID. IEEE Trans. Comput. Soc. Syst..

[42] Dimitriadou E., Lanitis A. (2023). Student action recognition for improving teacher feedback during tele-education. IEEE Trans. Learn. Technol..

[43] Foster J., Korban M., Youngs P., Watson G., Acton S. (2024). Automatic classification of activities in classroom videos. Comput. Educ. Artif. Intell..

[44] Pabba C., Bhardwaj V., Kumar P. (2024). A visual intelligent system for students’ behavior classification using body pose and facial features in a smart classroom. Multimed. Tools Appl..

[45] Keras TimeDistributed Layer. https://keras.io/api/layers/recurrent_layers/time_distributed/.

[46] Baldi P., Sadowski P., Burges C., Bottou L., Welling M., Ghahramani Z., Weinberger K. (2013). Understanding Dropout. Proceedings of the Advances in Neural Information Processing Systems.

[47] Keras Recurrent Layers. https://keras.io/api/layers/recurrent_layers/.

[48] Tran D., Bourdev L., Fergus R., Torresani L., Paluri M. Learning spatiotemporal features with 3D convolutional networks. Proceedings of the IEEE International Conference on Computer Vision (ICCV).

[49] Ying X. (2019). An overview of overfitting and its solutions. IOP J. Phys. Conf. Ser..

[50] Dosovitskiy A., Beyer L., Kolesnikov A., Weissenborn D., Zhai X., Unterthiner T., Dehghani M., Minderer M., Heigold G., Gelly S. (2020). An image is worth 16x16 words: Transformers for image recognition at scale. arXiv.

[51] Shi X., Chen Z., Wang H., Yeung D.Y., Wong W.K., Woo W.C. (2015). Convolutional LSTM network: A machine learning approach for precipitation nowcasting. Adv. Neural Inf. Process. Syst..

[52] Berrar D., Ranganathan S., Gribskov M., Nakai K., Schönbach C. (2019). Cross-Validation. Encyclopedia of Bioinformatics and Computational Biology.

[53] Fushiki T. (2011). Estimation of prediction error by using K-fold cross-validation. Stat. Comput..

[54] Lee D., In J., Lee S. (2015). Standard deviation and standard error of the mean. Korean J. Anesthesiol..

[55] Wikipedia Confusion Matrix. https://en.wikipedia.org/wiki/Confusion_matrix.

[56] Liashchynskyi P., Liashchynskyi P. (2019). Grid search, random search, genetic algorithm: A big comparison for NAS. arXiv.

[57] Wikipedia Learning Rate. https://en.wikipedia.org/wiki/Learning_rate.

[58] Keras Optimizers. https://keras.io/api/optimizers/.

[59] Kim T. (2015). T test as a parametric statistic. Korean J. Anesthesiol..

[60] Keras Documentation GaussianNoise Layer. https://keras.io/api/layers/regularization_layers/gaussian_noise/.

[61] Trabelsi Z., Alnajjar F., Parambil M., Gochoo M., Ali L. (2023). Real-time attention monitoring system for classroom: A deep learning approach for student’s behavior recognition. Big Data Cogn. Comput..

[62] Almusaed A., Almssad A., Yitmen I., Homod R. (2023). Enhancing student engagement: Harnessing “AIED”’s power in hybrid education—A review analysis. Educ. Sci..

[63] Thabtah F., Hammoud S., Kamalov F., Gonsalves A. (2020). Data imbalance in classification: Experimental evaluation. Inf. Sci..

[64] Wan S., Qi L., Xu X., Tong C., Gu Z. (2020). Deep learning models for real-time human activity recognition with smartphones. Mob. Netw. Appl..

[65] Zhang C., Bengio S., Hardt M., Recht B., Vinyals O. (2021). Understanding deep learning (still) requires rethinking generalization. Commun. ACM.

[66] Ahn S., Kim S., Ko J., Yun S.Y. Fine-tuning pre-trained models for robustness under noisy labels. Proceedings of the Thirty-Third International Joint Conference on Artificial Intelligence.

[67] Levi N., Bloch I.M., Freytsis M., Volansky T. (2022). Noise Injection Node Regularization for Robust Learning. arXiv.

[68] Phan N., Wu X., Hu H., Dou D. (2017). Adaptive Laplace mechanism: Differential privacy preservation in deep learning. Proceedings of the 2017 IEEE International Conference on Data Mining (ICDM).

[69] Dutta S., Burk J., Santer R., Zwiggelaar R., Boongoen T. (2023). Noise profiling for ANNs: A bio-inspired approach. Proceedings of the UK Workshop on Computational Intelligence.

[70] Jospin L., Laga H., Boussaid F., Buntine W., Bennamoun M. (2022). Hands-on Bayesian neural networks—A tutorial for deep learning users. IEEE Comput. Intell. Mag..

[71] Thakur U., Vidyarthi A., Prajapati A. (2024). Recognition of real-time video activities using stacked Bi-GRU with fusion-based deep architecture. J. Univers. Comput. Sci..

[72] Bocus M., Li W., Vishwakarma S., Kou R., Tang C., Woodbridge K., Craddock I., McConville R., Santos-Rodriguez R., Chetty K. (2022). OPERAnet, a multimodal activity recognition dataset acquired from radio frequency and vision-based sensors. Sci. Data.

[73] Wang J., Jiang H., Liu Y., Ma C., Zhang X., Pan Y., Liu M., Gu P., Xia S., Li W. (2024). A comprehensive review of multimodal large language models: Performance and challenges across different tasks. arXiv.

[74] Motamedi E., Behzad K., Zandi R., Salehinejad H., Siami M. (2024). Robustness evaluation of machine learning models for robot arm action recognition in noisy environments. Proceedings of the ICASSP 2024 IEEE International Conference on Acoustics, Speech and Signal Processing (ICASSP).

[75] Burgon A., Sahiner B., Petrick N., Pennello G., Cha K., Samala R. (2024). Decision region analysis for generalizability of artificial intelligence models: Estimating model generalizability in the case of cross-reactivity and population shift. J. Med. Imaging.

